# Phase 1 trial of HR070803 (an Irinotecan liposome) in combination with 5-fluorouracil, leucovorin, and oxaliplatin for untreated advanced or metastatic pancreatic ductal adenocarcinoma

**DOI:** 10.1186/s12916-025-04234-4

**Published:** 2025-07-07

**Authors:** Qiang Xu, Xue Zhao, Xianze Wang, Ruizhe Zhu, Yuejuan Cheng, Tao Xia, Heshui Wu, He Tian, Yuping Sun, Mingjun Zhang, Chuntao Gao, Deliang Fu, Xiaojie Wu, Tongsen Zheng, Xiaoyu Yin, Yili Chen, Xiaobing Chen, Zhihua Li, Rufu Chen, Xue Yang, Huan Wang, Quanren Wang, Xiaohong Han, Wenming Wu

**Affiliations:** 1 General Surgery Department, Dongcheng District, Peking Union Medical College Hospital, No. 1 Shuaifuyuan, Beijing, 100730 China; 2 Clinical Pharmacology Research Centre, Dongcheng District, Peking Union Medical College Hospital, No. 1 Shuaifuyuan, Beijing, 100730 China; 3 Department of Medical Oncology, Peking Union Medical College Hospital, Beijing, China; 4 Division of Gastrointestinal and Pancreatic Surgery, Zhejiang Provincial People’s Hospital, Hangzhou, China; 5 Pancreatic Surgery, Union Hospital Tongji Medical College, Huazhong University of Science and Technology, Wuhan, China; 6 Second Ward of Internal Medicine Digestive Disease, Shandong Cancer Hospital and Institute, Shandong First Medical University and Shandong Academy of Medical Sciences, Jinan, China; 7 Phase I Clinical Research Center, Shandong Cancer Hospital and Institute, Shandong First Medical University and Shandong Academy of Medical Sciences, Jinan, China; 8 Department of Oncology, The Second Affiliated Hospital of Anhui Medical University, Hefei, China; 9 Pancreatic Surgery, Tianjin Medical University Cancer Institute & Hospital, Tianjin, China; 10 Pancreatic Surgery, Huashan Hospital, Fudan University, Shanghai, China; 11 Phase I Clinical Research Laboratory, Huashan Hospital, Fudan University, Shanghai, China; 12 Phase I, Clinical Research Laboratory, Harbin Medical University Cancer Hospital, Harbin, China; 13 Department of Pancreatobiliary Surgery, The First Affiliated Hospital, Sun Yat-Sen University, Guangzhou, China; 14 Phase I Clinical Trial Center, The First Affiliated Hospital, Sun Yat-Sen University, Guangzhou, China; 15 Department of Oncology, The Affiliated Cancer Hospital, Zhengzhou University, Zhengzhou, China; 16 Department of Oncology, Sun Yat-Sen Memorial Hospital, Sun Yat-Sen University, Guangzhou, China; 17 Pancreatic Center, Guangdong Provincial People’s Hospital, Guangzhou, China; 18 Jiangsu Hengrui Pharmaceuticals Co., Ltd, Shanghai, China

**Keywords:** HR070803, liposomal irinotecan, untreated metastatic PDAC, phase 1 trial, RP2D

## Abstract

**Background:**

This study assessed the safety, preliminary antitumor activity, and pharmacokinetics of HR070803 (a novel liposomal irinotecan) in combination with 5-FU/LV and oxaliplatin for treatment-naive patients with unresectable locally advanced or metastatic pancreatic ductal adenocarcinoma (PDAC).

**Methods:**

This multicenter, open-label, single-arm, dose-escalation phase 1 study recruited treatment-naive patients aged 18–70 years with unresectable locally advanced or metastatic PDAC. Treatment doses were escalated from 40/60 (HR070803 40 mg/m^2^ plus 5-FU/LV and oxaliplatin 60 mg/m^2^) to 60/60 and 60/85. The primary endpoints were maximum tolerated dose (MTD) and the recommended phase 2 dose (RP2D). Secondary endpoints included safety, preliminary antitumor activity, and pharmacokinetics.

**Results:**

A total of 41 patients were enrolled, including 6, 17, and 18 patients in the 40/60, 60/60, and 60/85 group, respectively. Only one patient in the 60/60 group experienced dose-limiting toxicities of grade 3 increased alanine aminotransferase and grade 3 increased aspartate aminotransferase, and the MTD was not reached. Adverse events of grade ≥ 3 were reported in 31 (75.6%) patients, with the most common being decreased neutrophil count and increased gamma-glutamyltransferase. No treatment discontinuation occurred owing to adverse events, and there were no treatment-related deaths. The overall response (complete or partial response) rate was 29.3% in the total population. Pharmacokinetic results demonstrated prolonged circulation and slow release of free irinotecan.

**Conclusions:**

HR070803 plus 5-FU/LV and oxaliplatin demonstrated an acceptable toxicity, good antitumor activity, and favorable pharmacokinetic profile as a first-line treatment for patients with unresectable locally advanced or metastatic PDAC. Based on the comprehensive data obtained during the dose escalation and dose expansion stages, HR070803 60 mg/m^2^ plus 5-FU/LV and oxaliplatin 85 mg/m^2^ was chosen as the RP2D.

**Trial registration:**

Clinical trials.gov NCT04796948; registered March 15, 2021.

**Supplementary Information:**

The online version contains supplementary material available at 10.1186/s12916-025-04234-4.

## Background

Pancreatic ductal adenocarcinoma (PDAC) is one of the most lethal malignancies [1–4]. For those diagnosed with metastatic disease, chemotherapy remains the principal treatment while the 5-year survival rate is only about 3% [3, 5]. FOLFIRINOX (combination of irinotecan, 5-fluorouracil [5-FU], leucovorin [LV], and oxaliplatin) is recommended as one of the first-line regimens by most guidelines [6–8]. Although the objective response rate of FOLFIRINOX may reach up to 31.6%, its application is limited by a high frequency of adverse events such as neutropenia, febrile neutropenia, diarrhea, and neuropathy [6–8].

Irinotecan has been widely used in a variety of cancers, and SN-38 is the active metabolite [9–11]. Liposomal irinotecan, which employed liposome encapsulation to maintain the concentration of irinotecan and SN-38 within tumor, was developed to promote drug delivery, reduce drug-related toxicities, and enhance anti-tumor efficacy [12–15]. Based on the NAPOLI-1 trial [16], liposomal irinotecan (Onivyde®) in combination with 5-FU/LV has been approved for mPDAC following progression with gemcitabine-based therapy. Furthermore, the NAPOLI-3 trial demonstrated that the NALIRIFOX (liposomal irinotecan plus 5-FU/LV and oxaliplatin) exhibited significant improvements in overall survival and progression-free survival compared to nab-paclitaxel plus gemcitabine in patients with mPDAC, leading to its approval for mPDAC in the first-line treatment setting [17]. However, the high mortality rate and poor prognosis associated with mPDAC underscore the need for novel agents with enhanced antitumor activity and low toxicity in addressing this unmet medical requirement for the patient population.

HR070803, a novel liposomal irinotecan, utilizes a new nano-liposomal formulation incorporating surface-modified PEG-phospholipids with a particle size of approximately 80–90 nM, has been developed for treating tumor since 2008. This formulation effectively shields the recognition and uptake by the reticuloendothelial system, ensuring high drug loading and stability [18, 19]. Nano-liposomal formulation could enhance the permeability and retention effect of the drug, prolong drug exposure time in tumor tissue, and facilitate targeted delivery to tumor tissues [20–22]. Additionally, this formula has the potential to improve the therapeutic index of irinotecan by reducing the maximum plasma concentration (C_max_), thereby mitigating dose-related side effects [12–14]. A randomized phase 3 study demonstrated that HR070803 (56.5 mg/m^2^) in combination with 5-FU/LV significantly improved the median overall survival compared to placebo in combination with 5-FU/LV, and this combination has been approved for use in locally advanced or metastatic pancreatic cancer following gemcitabine therapy failure in China [23].

Here, we reported results from a multicenter, open-label, phase 1, dose-escalation and expansion study conducted to assess the safety, preliminary antitumor activity, and pharmacokinetics of HR070803 in combination with 5-FU/LV and oxaliplatin in treatment-naive patients with unresectable locally advanced or metastatic PDAC.

## Methods

### Patients

Eligible participants were aged 18–70 years with histologically or cytologically confirmed unresectable locally advanced or metastatic PDAC, had not received systemic antitumor treatment, had at least one measurable target lesion according to Response Evaluation Criteria in Solid Tumors (RECIST) v1.1 criteria, had an Eastern Cooperative Oncology Group performance status of 0 or 1, had adequate hematological, hepatic, renal, and cardiac function and a life expectancy of ≥ 3 months. Patients who had received the last dose of adjuvant chemotherapy > 12 months before screening and who had recovered from the toxicities of adjuvant therapy (grade ≤ 1) were eligible. Key exclusion criteria included pancreatic cancers originating from non-pancreatic ductal epithelium, known central nervous system metastasis, and harboring homozygous mutations in the UGT1A1*28/*6 gene.

### Study design and procedures

This multicenter, open-label, single-arm, phase 1 study (NCT04796948) was conducted in 12 sites in China (Additional file 1: Table S1) and consisted of a dose escalation stage and a dose expansion stage.

In the dose escalation stage, a traditional "3 + 3" design was implemented and the dose-escalation scheme was designed as below: 40/60 group, HR070803 40 mg/m^2^ (by free-base, the same below) plus 5-FU/LV and oxaliplatin 60 mg/m^2^; 60/60 group, HR070803 60 mg/m^2^ plus 5-FU/LV and oxaliplatin 60 mg/m^2^; and 60/85 group, HR070803 60 mg/m^2^ plus 5-FU/LV and oxaliplatin 85 mg/m^2^. The starting dose, dosing frequency, and maximum dose of HR070803 and oxaliplatin were determined based on animal toxicity studies and a phase 1b clinical trial of HR070803 (data on file, Hengrui), as well as the results of a similar drug, FOLFIRINOX, in the treatment of solid tumors [24]. Patients were administered via intravenous infusion with the order as follows: oxaliplatin over 2 h followed by HR070803 over 90 (± 5) minutes, then, fixed dose of LV (400 mg/m^2^ in all cohorts) over 30 min followed by 5-FU (2400 mg/m^2^ in all cohorts) over 46–48 h every 2 weeks (q2w), on days 1 and 15 of each 4-week cycle.

The following adverse events that occurred during cycle 1 in the dose escalation stage and were related to any of the treatment components were considered as dose-limiting toxicity: grade 3 or 4 non-hematological toxicity (except for nausea, vomiting, or diarrhea without treatment); grade 4 neutropenia lasting for ≥ 5 days or febrile neutropenia; grade 3 thrombocytopenia with bleeding or grade 4 thrombocytopenia; grade 4 anemia; or any treatment-related toxicity leading to dose delay for ≥ 15 days. If dose-limiting toxicities were experienced by ≥ 2 patients in 40/60 group, dose de-escalation would occur to a lower dose of HR070803 (i.e. 30 mg/m^2^). If no dose-limiting toxicity was observed in the 60/85 group, an additional dose-escalation cohort could be added following the recommendation of the safety monitor committee (SMC). The maximum tolerated dose was defined as the HR070803 dose below which dose-limiting toxicity occurred in ≥ 2 patients among the initially enrolled 3 patients or ≥ 2 patients among the initially enrolled 6 patients in a cohort experienced a dose-limiting toxicity during days 1 to 28 in the first treatment cycle.

Following the completion of the dose escalation stage, the dose expansion stage would be initiated based on the recommendations of the SMC to recruit additional participants to further evaluate the safety, pharmacokinetics, and preliminary efficacy. The treatment schedule and recommended dose level in the dose expansion stage were based on a thorough review and evaluation of all available data from the dose escalation stage by the SMC. The recommended phase 2 dose (RP2D) was subsequently decided by the SMC based on the comprehensive data obtained during the dose escalation and dose expansion stages, including the safety and tolerability, pharmacokinetics, and preliminary antitumor efficacy.

The protocol and all amendments were approved by the ethics committee of each study center. The study was conducted in accordance with the Declaration of Helsinki and Good Clinical Practice guidelines. All patients provided written, informed consent.

### Assessments

Adverse events were monitored from treatment initiation to 28 days after the last dose and were graded according to National Cancer Institute Common Terminology Criteria for Adverse Events (NCI-CTCAE) v5.0. Tumor response was evaluated every 8 weeks (± 7 days) after the first treatment using computed tomography or magnetic resonance imaging according to RECIST v1.1, until progression disease, new antitumor therapy initiation, withdrawal of consent, lost follow-up or death, whichever occurred first. Complete or partial responses were required to be confirmed ≥ 4 weeks after the first documentation.

For pharmacokinetic assessment, blood samples (4 mL) were collected from each patient in the dose escalation and dose expansion cohorts at 12 timepoints (pre-dose, 1.5 h, 2 h, 4 h, 8 h, 12 h, 24 h, 36 h, 48 h, 72 h, 96 h, and 120 h) after the first dose of HR070803 infusion in cycle 1. Plasma concentrations of total irinotecan, free irinotecan, and SN-38 were quantitated by validated liquid chromatography-tandem mass spectrometry analytical methods.

### Outcomes

The primary endpoints were to explore the maximum tolerated dose and the RP2D. Secondary endpoints included: safety, objective response rate, disease control rate, duration of response, progression-free survival, overall survival, and pharmacokinetics. Pharmacokinetic parameters included: C_max_, time to C_max_ (T_max_), the area under the plasma concentration-time curve (AUC_last_ and AUC_inf_), half-life of drug elimination (t_1/2_), clearance (CL), and the volume of distribution (V_z_).

### Statistical analyses

No formal hypothesis testing was implemented in this study. For dose escalation stage, the number of patients enrolled was dependent on the actual number of dose-limiting toxicities observed. A total of sample size of 40–52 patients was estimated for this study.

Safety was assessed in the safety set, which included all patients who received at least one administration with at least one post-baseline safety record. Efficacy was analyzed in the full analysis set, where all enrolled patients with at least one administration were included. Plasma concentrations and pharmacokinetic parameters were analyzed in patients who received at least one administration and had at least one post-baseline evaluable pharmacokinetic data. The median progression-free survival, overall survival, and duration of response were analyzed using the Kaplan-Meier method with 95% confidence intervals (CIs) calculated using Brookmeyer-Crowley method. Clopper-Pearson method was utilized to calculate 95% CIs for objective response rate and disease control rate. Baseline characteristics, toxicities, and certain pharmacokinetic parameters were summarized descriptively. Continuous variables were summarized as mean (standard deviation [SD]) or median (interquartile range [IQR] or range), while categorical variables were described by frequency (percentage, %). The plasma concentration versus time profiles were plotted. Non-compartment model (Phoenix WinNonlin v8.1 or higher, Certara, LP, Princeton, NJ, USA) was employed to calculate certain pharmacokinetic parameters. All the statistical analyses were performed using SAS version 9.4.

## Results

### Patients and baseline characteristics

**Fig. 1:**
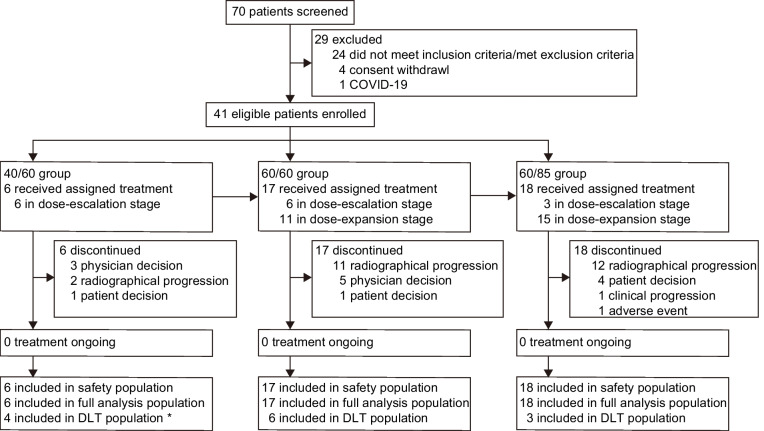
Patient disposition. Following the evaluation of the tolerability and antitumor activity in the first two stages, we decided to expand the number of patients in the dose expansion stage, and no further enrollment of patients in the clinical expansion stage was conducted. *Among the first 3 patients enrolled in the 40/60 dose group, one patient had an adverse event (hepatic function abnormality of grade 3, a suspected dose-limiting toxicity) that was initially considered a dose-limiting toxicity. Following the “3 + 3” rule, we subsequently enrolled another 3 patients in the 40/60 group. However, upon follow-up assessment, it was found that hepatic function abnormality of grade 3 was caused by the obstruction of the biliary tract induced by pancreatic tumor, and was not a dose-limiting toxicity related to the treatment. Therefore, there was no occurrence of dose-limiting toxicity in the 40/60 group. The dose-limiting toxicity analysis set included all patients who have been enrolled in the dose escalation stage, have received at least one dose of the study drug, and have completed dose-limiting toxicity assessment. There were two patients enrolled in the 40/60 group during the dose-escalation stage did not meet the criteria of the dose-limiting toxicity analysis set. One patient had biliary stricture due to tumor-related reasons, which made it impossible to complete the dose-limiting toxicity observation. The other patient voluntarily requested to terminate the study treatment and thus did not complete the dose-limiting toxicity observation. Abbreviation: DLT, dose-limiting toxicity

Between April 8, 2021 and November 27, 2022, 41 patients were enrolled in the dose escalation (*n* = 15) and the dose expansion group (*n* = 26) from 12 sites in China. In the dose escalation stage, there were 6 patients enrolled in the 40/60 group, 6 in the 60/60 group, and 3 in the 60/85 group. In the dose expansion stage, 11 patients were enrolled in the 60/60 group and 15 were in the 60/85 group (Fig. 1). The median age of the study group was 57 years and 73.2% of them were male. Five (12.2%) patients were diagnosed as stage III and 36 (87.8%) patients were diagnosed as stage IV. For patients with metastatic diseases, liver (*n* = 30, 73.2%) was the most frequent metastatic site and 24 (58.5%) patients have more than 2 metastases (Table 1). As of the data cutoff on June 28, 2024, the median treatment duration was 3.6 (IQR 0.1–9.0) months in 40/60 group, 3.4 (IQR 1.0–7.0) months in 60/60 group, and 3.4 (IQR 1.6–4.6) months in 60/85 group; the median follow-up duration was 21.7 (IQR 21.2–23.4) months. All patients discontinued the treatment with radiographical progression being the primary reason (*n* = 25, 61.0%), and 29 (70.7%) patients received post-discontinuation systemic antitumor therapy (Additional file 1: Table S2).

**Table 1:** Baseline characteristics

	**40/60 group (N = 6)**	**60/60 group (N = 17)**	**60/85 group (N = 18)**	**Total (N = 41)**
Age, years	56.0 (38–66)	57.0 (27–68)	58.0 (36–67)	57.0 (27–68)
Male	5 (83.3)	12 (70.6)	13 (72.2)	30 (73.2)
ECOG performance score				
0	1 (16.7)	5 (29.4)	4 (22.2)	10 (24.4)
1	5 (83.3)	12 (70.6)	14 (77.8)	31 (75.6)
Disease stage at initial diagnosis				
III	0	1 (5.9)	4 (22.2)	5 (12.2)
IV	6 (100)	16 (94.1)	14 (77.8)	36 (87.8)
Pancreatic tumor location				
Head	1 (16.7)	5 (29.4)	8 (44.4)	14 (34.1)
Other	5 (83.3)	12 (70.6)	10 (55.6)	27 (65.9)
Number of sites with metastasis				
< 2	2 (33.3)	5 (29.4)	8 (44.4)	15 (36.6)
≥ 2	4 (66.7)	11 (64.7)	9 (50.0)	24 (58.5)
Site of metastases				
Liver	6 (100.0)	13 (76.5)	11 (61.1)	30 (73.2)
Lung	1 (16.7)	2 (11.8)	2 (11.1)	5 (12.2)
Regional lymph node	2 (33.3)	5 (29.4)	9 (50.0)	16 (39.0)
Distant lymph node	0	5 (29.4)	3 (16.7)	8 (19.5)
Peritoneum	0	0	1 (5.6)	1 (2.4)
Other	1 (16.7)	4 (23.5)	3 (16.7)	8 (19.5)
CA19-9, U/mL	834.0 (2.0–2483.0)	177.0 (8.1–20,481.5)	980.0 (2.0–12000.0)	316.9 (2.0–20481.5)
UGT1A1*28 genotype				
TA6/7	0	3 (17.6)	4 (22.2)	7 (17.1)
TA6/6	6 (100)	14 (82.4)	14 (77.8)	34 (82.9)
UGT1A1*6 genotype				
G/G	4 (66.7)	12 (70.6)	8 (44.4)	24 (58.5)
A/G	2 (33.3)	5 (29.4)	10 (55.6)	17 (41.5)

Data are median (range) or n (%). ECOG, Eastern Cooperative Oncology Group

### Tolerability and safety

In the dose escalation stage, one patient in the 60/60 group experienced dose-limiting toxicities of grade 3 increased alanine aminotransferase and grade 3 increased aspartate aminotransferase, which recovered spontaneously without intervention. Therefore, the maximum tolerated dose was not reached.

Safety was assessed in 41 patients, with all of them experiencing at least one adverse events (Table 2). The most frequently reported AEs included neutropenia (82.9%, 34/41), nausea (80.5%, 33/41), decreased white blood cell (70.7%, 29/41), and anaemia (70.7%, 29/41). Adverse events of grade ≥ 3 were reported in 31 (75.6%) patients, with the most common ones being decreased neutrophil count (41.5%, 17/41), increased gamma-glutamyltransferase (19.5%, 8/41), white decreased blood cell count (14.6%, 6/41), and increased alanine aminotransferase (12.2%, 5/41). Treatment-related adverse events of grade ≥ 3 were reported in 25 (61.0%) patients, and the most common ones were decreased neutrophil count (41.5%, 17/41), increased gamma-glutamyltransferase (19.5%, 8/41), and decreased white blood cell (14.6%, 6/41) (Additional file 1: Table S3). Serious adverse events occurred in 19 (46.3%) patients, and those in 8 patients (19.5%) were considered to be treatment-related (Additional file 1: Table S4). Dose reduction and treatment interruption of any treatment component owing to adverse event was reported in 8 (19.5%) patients and 25 (61.0%) patients, respectively. No treatment discontinuation occurred due to adverse events, and no deaths attributed to adverse events were deemed treatment-related.

**Table 2:** Adverse events

	40/60 group (N = 6)		60/60 group (N = 17)		60/85 group (N = 18)		Total (N = 41)	
	Any grade	Grade 3–5	Any grade	Grade 3–5	Any grade	Grade 3–5	Any grade	Grade 3–5
Any	6 (100)	5 (83.3)	17 (100)	13 (76.5)	18 (100)	13 (72.2)	41 (100)	31 (75.6)
Neutrophil count decreased	3 (50.0)	2 (33.3)	16 (94.1)	6 (35.3)	15 (83.3)	9 (50.0)	34 (82.9)	17 (41.5)
Nausea	6 (100)	0	13 (76.5)	1 (5.9)	14 (77.8)	0	33 (80.5)	1 (2.4)
White blood cell count decreased	3 (50.0)	1 (16.7)	13 (76.5)	1 (5.9)	13 (72.2)	4 (22.2)	29 (70.7)	6 (14.6)
Anaemia	1 (16.7)	0	13 (76.5)	1 (5.9)	15 (83.3)	0	29 (70.7)	1 (2.4)
Alanine aminotransferase increased	4 (66.7)	0	9 (52.9)	3 (17.6)	12 (66.7)	2 (11.1)	25 (61.0)	5 (12.2)
Diarrhoea	3 (50.0)	0	10 (58.8)	1 (5.9)	10 (55.6)	0	23 (56.1)	1 (2.4)
Aspartate aminotransferase increased	3 (50.0)	0	9 (52.9)	2 (11.8)	10 (55.6)	2 (11.1)	22 (53.7)	4 (9.8)
Gamma-glutamyltransferase increased	4 (66.7)	1 (16.7)	10 (58.8)	2 (11.8)	7 (38.9)	5 (27.8)	21 (51.2)	8 (19.5)
Platelet count decreased	3 (50.0)	0	7 (41.2)	0	11 (61.1)	0	21 (51.2)	0
Decreased appetite	2 (33.3)	0	8 (47.1)	0	8 (44.4)	1 (5.6)	18 (43.9)	1 (2.4)
Constipation	3 (50.0)	0	7 (41.2)	0	7 (38.9)	0	17 (41.5)	0
Hyponatraemia	1 (16.7)	0	5 (29.4)	0	10 (55.6)	0	16 (39.0)	0
Vomiting	1 (16.7)	0	7 (41.2)	0	8 (44.4)	0	16 (39.0)	0
Hypoalbuminaemia	0	0	8 (47.1)	0	7 (38.9)	0	15 (36.6)	0
Weight decreased	1 (16.7)	0	5 (29.4)	0	9 (50.0)	1 (5.6)	15 (36.6)	1 (2.4)
Hypokalaemia	0	0	5 (29.4)	1 (5.9)	9 (50.0)	3 (16.7)	14 (34.1)	4 (9.8)
Asthenia	2 (33.3)	0	6 (35.3)	1 (5.9)	5 (27.8)	0	13 (31.7)	1 (2.4)
Abdominal pain	3 (50.0)	0	7 (41.2)	0	2 (11.1)	0	12 (29.3)	0
Blood alkaline phosphatase increased	0	0	5 (29.4)	0	6 (33.3)	0	11 (26.8)	0
COVID-19	0	0	2 (11.8)	0	9 (50.0)	0	11 (26.8)	0
Pyrexia	0	0	6 (35.3)	0	4 (22.2)	0	10 (24.4)	0
Hyperglycaemia	0	0	6 (35.3)	0	3 (16.7)	0	9 (22.0)	0
Lymphocyte count decreased	1 (16.7)	1 (16.7)	4 (23.5)	0	4 (22.2)	2 (11.1)	9 (22.0)	3 (7.3)

Data are n (%). Adverse events of any grade occurring in more than 20% of total patients and adverse events of grade 3–5 occurring in more than 5% of total patients were listed

### Antitumor activity

**Fig. 2:**
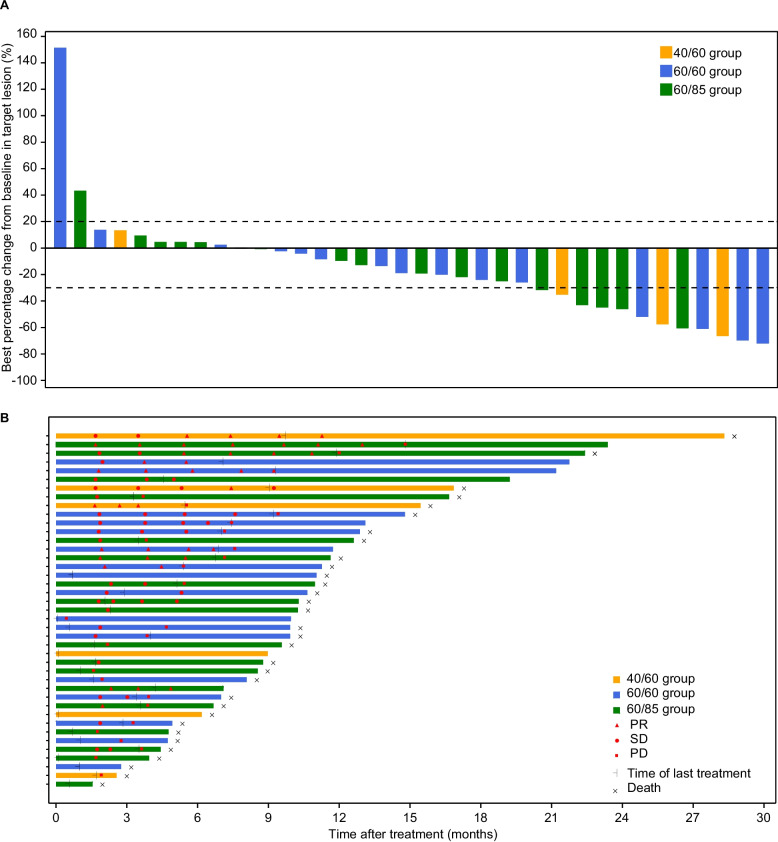
Tumor response. **A** The best percentage change from baseline in target tumor lesion size. Each bar represents a patient. **B** Duration of tumor response in the full analysis population. PR, partial response; SD, stable disease; PD, progression disease

All of the 41 patients were included in the full analysis set. The confirmed overall response (complete or partial response) was achieved in 10 (24.4%, 95% CI 12.4%–40.3%) patients, including 2 (33.3%) patients from 40/60 group, 4 (23.5%) from 60/60 group, and 4 (22.2%) from 60/85 group (Additional file 1: Table S5). Disease control was achieved in 28 (68.3%, 95% CI 51.9%–81.9%) patients. For patients who achieved complete or partial responses, the estimated median duration of response was 5.5 (95% CI 2.0–13.2) months (Additional file 1: Fig. S1A). The tumor response and response duration for each patient are presented in the waterfall plot (Fig. 2A) and swimmer plot (Fig. 2B). As of the data cutoff, 28 (68.3%) of the 41 patients experienced PFS events. The median progression-free survival was 5.4 (95% CI 3.7–7.2) months, and the 6-months progression-free survival rate was 41.4% (95% CI 24.5%–57.5%) (Additional file 1: Fig. S1B). Thirty-six (87.8%) death events occurred, and the median overall survival data was 10.3 (95% CI 8.2–11.2) months (Additional file 1: Fig. S1C).

### Pharmacokinetics

**Fig. 3:**
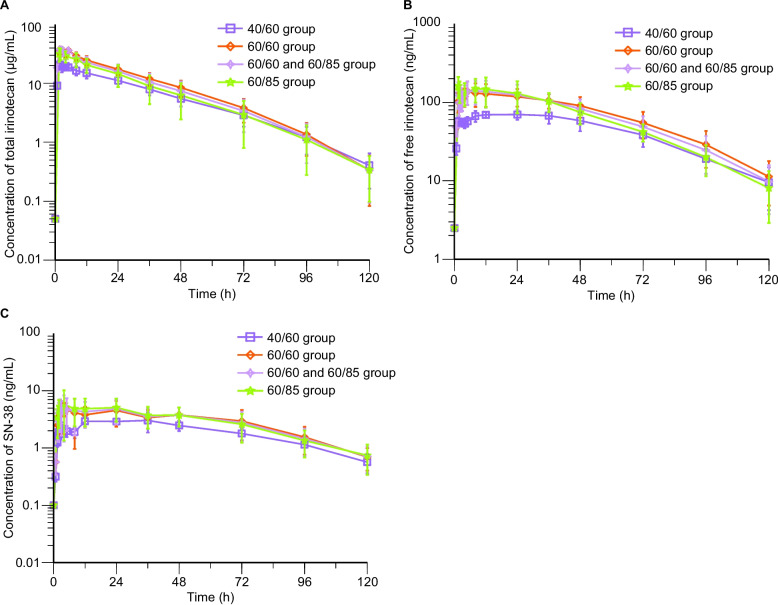
Plasma concentration-time profiles of irinotecan and its metabolite SN-38. **A** Total irinotecan. **B** Free irinotecan. **C** SN-38

Thirty-eight patients were included for plasma drug concentration analysis and 37 were included for pharmacokinetic parameters analysis. After a single administration of HR070803, the peak plasma concentrations of total irinotecan, free irinotecan, and SN-38 in the 40/60, 60/60, and 60/85 groups were reached within 1.75–1.98 h, 1.55–24.11 h, and 12.00–24.00 h, respectively. The exposure (C_max_, AUC_last_, and AUC_inf_) of total irinotecan, free irinotecan, and SN-38 increased with an increasing administration dose, with level of free irinotecan exposure remarkable lower than that of the total irinotecan. The half-life of drug elimination of total irinotecan, free irinotecan, and SN-38 in the three groups was 14.49–18.53 h, 21.79–24.93 h, and 25.17–31.49 h, respectively. The clearance rate of total irinotecan among different dosage groups remained relatively consistent, ranging from 56.0 to 77.4 mL/(h*m^2^). The plasma concentration-time profiles of total irinotecan, free irinotecan, and SN-38 by dose are presented in Fig. 3, and pharmacokinetic parameters by dose are summarized in Table 3.

**Table 3:** Pharmacokinetic parameters

	**40/60 group**	**60/60 group**	**60/85 group**
Total irinotecan			
T_max_, h	1.75 (1.50–4.00)	1.76 (1.47–2.48)	1.98 (1.47–2.02)
C_max_, ug/mL	21.27 (3.08)	36.78 (4.03)	33.50 (7.43)
AUC_last_, h*ug/mL	709.13 (128.07)	1112.49 (235.6)	900.36 (358.51)
AUC_inf_, h*ug/mL	723.57 (133.27)	1122.43 (240.72)	909.52 (363.24)
t_1/2_, h	18.53 (4.45)	14.49 (3.49)	15.33 (3.45)
CL, mL/(h*m^2^)	57.17 (12.49)	56.00 (13.29)	77.44 (33.65)
V_z_, mL/m^2^	1489.85 (289.03)	1171.69 (395.98)	1611.91 (487.50)
Free irinotecan			
T_max_, h	24.11 (12.00–48.00)	8.00 (1.47–47.45)	1.55 (1.47–24.00)
C_max_, ng/mL	78.40 (8.44)	151.94 (38.23)	183.60 (56.82)
AUC_last_, h*ng/mL	5207.47 (902.17)	8486.49 (2217.24)	8005.41 (2121.53)
AUC_inf_, h*ng/mL	5619.02 (1078.98)	8922.17 (2360.31)	8365.61 (2108.39)
t_1/2_, h	24.93 (5.16)	22.39 (4.03)	21.79 (4.79)
SN-38			
T_max_, h	24.00 (11.93–36.00)	12.00 (2.02–48.00)	12.00 (3.98–36.25)
C_max_, ng/mL	3.45 (0.76)	6.20 (2.72)	7.29 (4.11)
AUC_last_, h*ng/mL	231.18 (36.19)	344.20 (117.00)	344.16 (120.36)
AUC_inf_, h*ng/mL	260.47 (49.31)	371.72 (124.89)	377.01 (129.67)
t_1/2_, h	31.49 (8.85)	25.17 (8.97)	29.71 (11.37)

Data are median (range) or mean (SD)

The evaluation of dose proportionality of plasma exposure to irinotecan and SN-38 was conducted using a power model. The levels of C_max_, AUC_last_, and AUC_inf_ of total irinotecan, free irinotecan, and SN-38 in plasma between the 60/60 group and 60/85 group (both irinotecan 60 mg/m^2^) were not significantly different (P > 0.05), suggesting that the 60/60 and 60/85 groups could be combined for analysis to indicate the dose of irinotecan at 60 mg/m^2^. The power model analysis showed that within the dose range of 40 to 60 mg/m^2^, the C_max_ of total irinotecan, free irinotecan, and SN-38 increased slightly higher than proportionally with dose; the AUC_last_ and AUC_inf_ of total irinotecan and SN-38 increased slightly less than proportionally with dose; and the AUC_last_ and AUC_inf_ of free irinotecan increased in a dose-dependent manner (Additional file 1: Fig. S2).

Based on the comprehensive data obtained during the dose escalation and dose expansion stages, including the safety and tolerability, pharmacokinetics, and preliminary antitumor efficacy, the SMC decided to select the highest dose in this study (HR070803 60 mg/m^2^ plus 5-FU/LV and oxaliplatin 85 mg/m^2^) as the RP2D.

## Discussion

In this phase 1 trial, we assessed the safety, preliminary antitumor activity, and pharmacokinetics of HR070803, a novel nano-liposomal formulation of irinotecan, in combination with 5-FU/LV plus oxaliplatin in treatment-naive patients with unresectable locally advanced or metastatic PDAC. The safety profile indicated that this treatment regimen was well-tolerated, with one patient from the 60/60 group experiencing dose-limiting toxicities of grade 3 increased alanine aminotransferase and increased aspartate aminotransferase, which spontaneously recovered within 8 days without intervention. The maximum tolerated dose was not reached. Based on safety, tolerability, pharmacokinetics, and preliminary antitumor efficacy, the SMC has decided to choose the highest dose in this study (HR070803 60 mg/m^2^ by free-base plus 5-FU/LV and oxaliplatin 85 mg/m^2^) as the RP2D. Pharmacokinetic results demonstrated a slower and more stable release of free irinotecan and SN-38 compared with conventional non-liposomal irinotecan [25].

The specificity of the most frequent adverse events was generally consistent with those expected for conventional non-liposomal irinotecan, and no new toxicity signals were identified [10, 26]. The slow-release nature of HR070803, owing to its nano-liposomal formula and distinctive preparation technology, helped maintain the peak concentrations of free irinotecan and SN-38 at lower and stable levels. This led to comparatively lower incidences of decreased neutrophil count (82.9% vs 54%–96.9%), diarrhea (56.1% vs 72.4%–88%), and cholinergic syndrome (2.4% vs 28.3%) compared to irinotecan hydrochloride [11, 26]. The adverse event profiles both in the total patients and the RP2D group (60/85) observed in the current trial were generally in line with that previously reported for liposome irinotecan [16, 17, 24]. Notably, no treatment discontinuations due to adverse events were observed, whereas the incidence of treatment discontinuation due to adverse events in the NAPOLI 3 study was 32% [17]. However, cross-trial comparisons should be interpreted with caution due to the lack of head-to-head studies.

The incidences of diarrhea at any grade were similar among the three groups, with only one case of grade ≥ 3 diarrhea occurring in the 60/60 group. This suggests that the dose escalation of HR070803 from 40 to 60 mg/m^2^ had little impact on the incidence of diarrhea. The incidence of grade ≥ 3 decreased neutrophil count remained stable with escalation of HR070803 dose from 40 to 60 mg/m^2^ but increased with oxaliplatin dose escalation from 60 to 85 mg/m^2^, indicating that it may be plausible to start with dose reduction of oxaliplatin when intolerable decreased neutrophil count occurs in subsequent phase 3 study. Oxaliplatin-containing regimens can potentially cause grade ≥ 3 sensory neuropathy (9.0% of patients receiving FOLFIRINOX in the PRODIGE 4 study) [6], but none was reported in our study. Therefore, in subsequent clinical studies with longer-term follow-up, it is necessary to continue monitoring whether the oxaliplatin in our combination strategy carries the risk of causing sensory neuropathy.

During the study, a significant COVID-19 outbreak occurred from November 2022 to January 2023, leading to medication administration delays for 13 patients in our study, 12 patients experienced a cumulative delay of ≥ 2 weeks. Notably, four patients had a cumulative delay in medication administration of ≥ 21 days before their initial tumor assessment. Considering the rapid progression of pancreatic cancer, these medication delays may have led to an underestimation of antitumor activity of the study treatment. Despite the impact of the COVID-19 pandemic on the study, 26 (63.4%) of the 41 patients achieved a reduction in tumor lesion size from baseline, with a percentage change of reduction ranging from 0.8% to 72.1%.

In terms of pharmacokinetics, the levels of free irinotecan exposure (AUC_last_ and AUC_inf_) in the 60/60 and 60/85 groups were only 0.76%–0.92% of the total irinotecan. Furthermore, when compared to free-form irinotecan [26], the administration of HR070803 resulted in higher overall exposure (C_max_, AUC_last_, and AUC_inf_) and a longer half-life of total irinotecan, as well as low C_max_ and prolonged half-life of free irinotecan. These findings suggest that HR070803 demonstrates characteristics of prolonged circulation and a slow release of irinotecan.

This study has several limitations. Firstly, the sample size was relatively small, which limited the precision of antitumor activity parameter estimates and made it infeasible to conduct subgroup analysis based on prognostic factors such as age, gender, disease stage, or biomarker status. Secondly, the study was also limited by its non-randomized design and the absence of a control group. Thirdly, due to the impact of the COVID-19 pandemic, the efficacy of the HR070803 combination regimen was underestimated. However, the primary significance of this study lies in the exploration of tolerable doses and the RP2D of this combination regimen in unresectable, locally advanced, or metastatic PDAC, and the efficacy results of this trial warrant further validation in a larger randomized study in this population.

## Conclusions

In conclusion, HR070803 plus 5-FU/LV and oxaliplatin demonstrated a tolerable safety profile with regard to both hematological and non-hematological adverse events and exhibited good antitumor activity as a first-line treatment for patients with unresectable, locally advanced, or metastatic PDAC. The slow but prolonged stable release of irinotecan and SN-38 due to the nanoscale liposome encapsulation further supports its potential efficacy and tolerability. A large randomized controlled phase 3 study comparing this combination regimen with gemcitabine/nab-paclitaxel in this population is currently underway.

## Supplementary Information

Additional file 1: Figures S1-S2 and Tables S1-S5. Fig. S1. Kaplan-Meier curves of efficacy endpoints. (A) Duration of response. (B) Progression-free survival. (C) Overall survival. Fig. S2. Dose proportionality of plasma exposure of total irinotecan, free irinotecan, and SN-38. (A) Total irinotecan. (B) Free irinotecan. (C) SN-38. Table S1. Participating sites. Table S2. Subsequent post-discontinuation antitumor therapy. Table S3. Treatment-related adverse events. Table S4. Serious adverse events. Table S5. Antitumor activity.

## Data Availability

Data are available from the corresponding authors upon reasonable request.

## Abbreviations

5-FU: 5-fluorouracil
AUC: The area under the plasma concentration-time curve
CI: Confidence interval
CL: Clearance
C_max_: Maximum plasma concentration
LV: Leucovorin
NCI-CTCAE: National Cancer Institute Common Terminology Criteria for Adverse Events
PDAC: Pancreatic ductal adenocarcinoma
RECIST: Response Evaluation Criteria in Solid Tumors
RP2D: Recommended phase 2 dose
SD: Standard deviation
SMC: Safety monitor committee
t_1/2_: Half-life of drug elimination
T_max_: Time to C_max_
V_z_: Volume of distribution
