# Supplementary material for: Phase 1 trial of HR070803 (an Irinotecan liposome) in combination with 5-fluorouracil, leucovorin, and oxaliplatin for untreated advanced or metastatic pancreatic ductal adenocarcinoma
Source: BMC Med. 2025 Jul 7;23:402. doi: 10.1186/s12916-025-04234-4 (PMC12232731; doi:10.1186/s12916-025-04234-4)
Supplement: Supplementary file 1 — Additional file 1: Figures S1-S2 and Tables S1-S5. Fig. S1. Kaplan-Meier curves of efficacy endpoints. (A) Duration of response. (B) Progression-free survival. (C) Overall survival. Fig. S2. Dose proportionality of plasma exposure of total irinotecan, free irinotecan, and SN-38. (A) Total irinotecan. (B) Free irinotecan. (C) SN-38. Table S1. Participating sites. Table S2. Subsequent post-discontinuation antitumor therapy. Table S3. Treatment-related adverse events. Table S4. Serious adverse events. Table S5. Antitumor activity. [file 12916_2025_4234_MOESM1_ESM.docx]

Additional File

Phase 1 Trial of HR070803 (an Irinotecan Liposome) in Combination with 5-Fluorouracil, Leucovorin, and Oxaliplatin for Untreated Advanced or Metastatic Pancreatic Ductal Adenocarcinoma

Qiang Xu, Xue Zhao, Xianze Wang, Ruizhe Zhu, Yuejuan Cheng, Tao Xia, Heshui Wu, He Tian, Yuping Sun, Mingjun Zhang, Chuntao Gao, Deliang Fu, Xiaojie Wu, Tongsen Zheng, Xiaoyu Yin, Yili Chen, Xiaobing Chen, Zhihua Li, Rufu Chen, Xue Yang, Huan Wang, Quanren Wang, Xiaohong Han, Wenming Wu

## Table of contents

[Table of contents 1](#_Toc166494997)

[Fig. S1. Kaplan-Meier curves of efficacy endpoints 2](#_Toc166494998)

[Fig. S2. Dose proportionality of plasma exposure of total irinotecan, free irinotecan, and SN-38 3](#_Toc166494999)

[Table S1. Participating sites 4](#_Toc166495000)

[Table S2. Subsequent post-discontinuation antitumor therapy 5](#_Toc166495001)

[Table S3. Treatment-related adverse events 6](#_Toc166495002)

[Table S4. Serious adverse events 8](#_Toc166495003)

[Table S5. Antitumor activity 9](#_Toc166495004)

## Fig. S1. Kaplan-Meier curves of efficacy endpoints

(A) Duration of response. (B) Progression-free survival. (C) Overall survival.


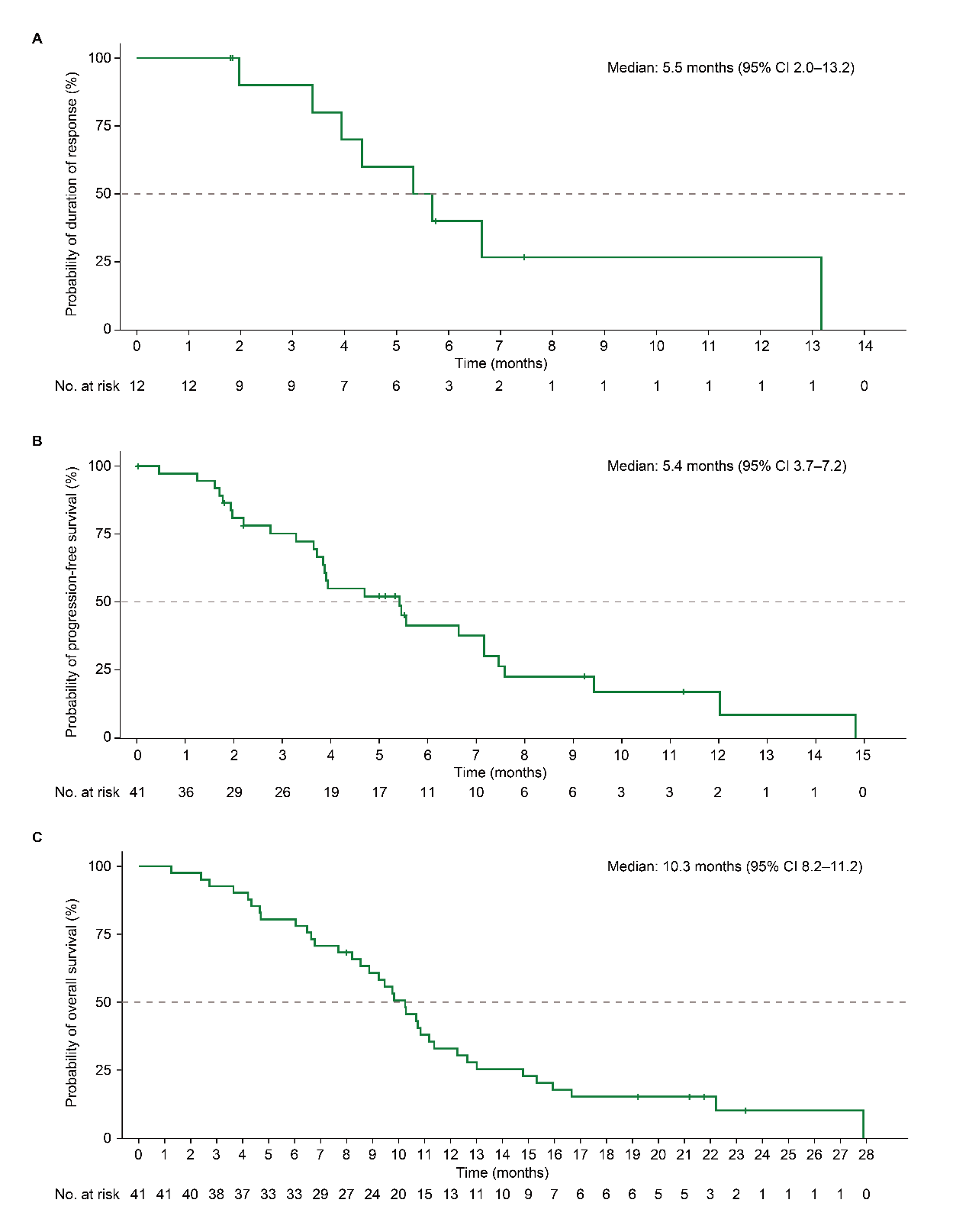


## Fig. S2. Dose proportionality of plasma exposure of total irinotecan, free irinotecan, and SN-38

(A) Total irinotecan. (B) Free irinotecan. (C) SN-38.


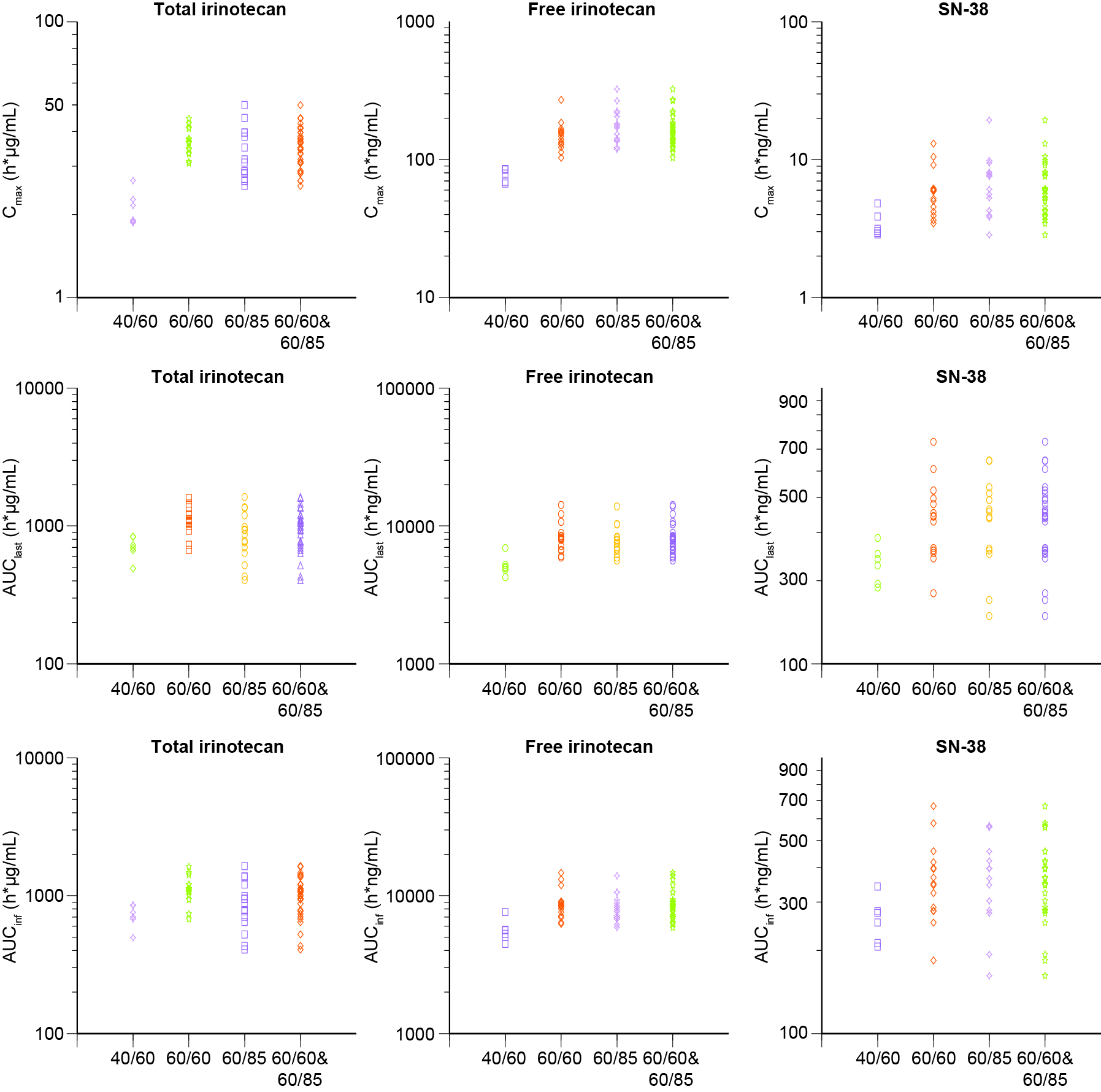


## Table S1. Participating sites

| Investigator | Study site | Number of patients |
| --- | --- | --- |
| Wenming Wu / Xiaohong Han | Peking Union Medical College Hospital | 16 |
| Yiping Mou / Xiangmin Tong | Zhejiang Provincial People's Hospital | 4 |
| Heshui Wu | Union Hospital Tongji Medical College Huazhong University of Science and Technology | 4 |
| Yuping Sun / He Tian | Shandong Cancer Hospital and Institute, Shandong First Medical University and Shandong Academy of Medical Sciences | 3 |
| Chuntao Gao | Tianjin Medical University Cancer Institute & Hospital | 3 |
| Mingjun Zhang | The Second Affiliated Hospital of Anhui Medical University | 3 |
| Deliang Fu / Xiaojie Wu | Huashan Hospital, Fudan University | 2 |
| Tongsen Zheng | Harbin Medical University Cancer Hospital | 2 |
| Xiaobing Chen | The Affiliated Cancer Hospital, Zhengzhou University | 1 |
| Rufu Chen | Guangdong Provincial People's Hospital | 1 |
| Zhihua Li | Sun Yat-sen Memorial Hospital, Sun Yat-sen University | 1 |
| Xiao-You Yin / Yili Chen | The First Affiliated Hospital, Sun Yat-sen University | 1 |

## Table S2. Subsequent post-discontinuation antitumor therapy

|  | 40/60 group (N=6) | 60/60 group (N=17) | 60/85 group (N=18) | Total (N=41) |
| --- | --- | --- | --- | --- |
| Systemic antitumor therapy | 5 (83.3) | 12 (70.6) | 12 (66.7) | 29 (70.7) |
| Chemotherapy | 5 (83.3) | 11 (64.7) | 10 (55.6) | 26 (63.4) |
| Targeted therapy | 1 (16.7) | 0 | 3 (16.7) | 4 (9.8) |
| Immunotherapy | 0 | 1 (5.9) | 5 (27.8) | 6 (14.6) |
| Other | 1 (16.7) | 0 | 0 | 1 (2.4) |

Data are n (%).

## Table S3. Treatment-related adverse events

|  | 40/60 group (N=6) | | 60/60 group (N=17) | | 60/85 group (N=18) | | Total (N=41) | |
| --- | --- | --- | --- | --- | --- | --- | --- | --- |
|  | Any grade | Grade 3–5 | Any grade | Grade 3–5 | Any grade | Grade 3–5 | Any grade | Grade 3–5 |
| Any | 6 (100) | 3 (50) | 17 (100) | 10 (58.8) | 18 (100) | 12 (66.7) | 41 (100) | 25 (61.0) |
| Neutrophil count decreased | 3 (50.0) | 2 (33.3) | 16 (94.1) | 6 (35.3) | 15 (83.3) | 9 (50.0) | 34 (82.9) | 17 (41.5) |
| Nausea | 6 (100) | 0 | 13 (76.5) | 1 (5.9) | 14 (77.8) | 0 | 33 (80.5) | 1 (2.4) |
| White blood cell count decreased | 3 (50.0) | 1 (16.7) | 13 (76.5) | 1 (5.9) | 13 (72.2) | 4 (22.2) | 29 (70.7) | 6 (14.6) |
| Anaemia | 1 (16.7) | 0 | 12 (70.6) | 1 (5.9) | 14 (77.8) | 0 | 27 (65.9) | 1 (2.4) |
| Alanine aminotransferase increased | 4 (66.7) | 0 | 8 (47.1) | 2 (11.8) | 12 (66.7) | 2 (11.1) | 24 (58.5) | 4 (9.8) |
| Diarrhoea | 3 (50.0) | 0 | 10 (58.8) | 1 (5.9) | 10 (55.6) | 0 | 23 (56.1) | 1 (2.4) |
| Gamma-glutamyltransferase increased | 4 (66.7) | 1 (16.7) | 10 (58.8) | 2 (11.8) | 7 (38.9) | 5 (27.8) | 21 (51.2) | 8 (19.5) |
| Platelet count decreased | 3 (50.0) | 0 | 7 (41.2) | 0 | 11 (61.1) | 0 | 21 (51.2) | 0 |
| Aspartate aminotransferase increased | 2 (33.3) | 0 | 8 (47.1) | 1 (5.9) | 10 (55.6) | 2 (11.1) | 20 (48.8) | 3 (7.3) |
| Decreased appetite | 2 (33.3) | 0 | 8 (47.1) | 0 | 7 (38.9) | 1 (5.6) | 17 (41.5) | 1 (2.4) |
| Vomiting | 1 (16.7) | 0 | 7 (41.2) | 0 | 8 (44.4) | 0 | 16 (39.0) | 0 |
| Asthenia | 2 (33.3) | 0 | 6 (35.3) | 1 (5.9) | 4 (22.2) | 0 | 12 (29.3) | 1 (2.4) |
| Hypoalbuminaemia | 0 | 0 | 5 (29.4) | 0 | 6 (33.3) | 0 | 11 (26.8) | 0 |
| Weight decreased | 0 | 0 | 2 (11.8) | 0 | 9 (50) | 1 (5.6) | 11 (26.8) | 1 (2.4) |
| Hyponatraemia | 0 | 0 | 3 (17.6) | 0 | 6 (33.3) | 0 | 9 (22.0) | 0 |
| Blood alkaline phosphatase increased | 0 | 0 | 3 (17.6) | 0 | 5 (27.8) | 0 | 8 (19.5) | 0 |
| Hypoaesthesia | 1 (16.7) | 0 | 3 (17.6) | 0 | 3 (16.7) | 0 | 7 (17.1) | 0 |
| Lymphocyte count decreased | 0 | 0 | 2 (11.8) | 0 | 4 (22.2) | 2 (11.1) | 6 (14.6) | 2 (4.9) |
| Constipation | 1 (16.7) | 0 | 3 (17.6) | 0 | 2 (11.1) | 0 | 6 (14.6) | 0 |
| Hypokalaemia | 0 | 0 | 0 | 0 | 5 (27.8) | 2 (11.1) | 5 (12.2) | 2 (4.9) |
| Blood bilirubin increased | 0 | 0 | 1 (5.9) | 0 | 4 (22.2) | 1 (5.6) | 5 (12.2) | 1 (2.4) |
| Alopecia | 1 (16.7) | 0 | 3 (17.6) | 0 | 1 (5.6) | 0 | 5 (12.2) | 0 |
| Proteinuria | 0 | 0 | 1 (5.9) | 0 | 4 (22.2) | 0 | 5 (12.2) | 0 |
| Bilirubin conjugated increased | 0 | 0 | 1 (5.9) | 0 | 1 (5.6) | 1 (5.6) | 2 (4.9) | 1 (2.4) |
| Infection | 0 | 0 | 1 (5.9) | 1 (5.9) | 1 (5.6) | 1 (5.6) | 2 (4.9) | 2 (4.9) |
| Intestinal obstruction | 0 | 0 | 1 (5.9) | 1 (5.9) | 1 (5.6) | 1 (5.6) | 2 (4.9) | 2 (4.9) |
| Eating disorder symptom | 0 | 0 | 1 (5.9) | 1 (5.9) | 0 | 0 | 1 (2.4) | 1 (2.4) |
| Pain | 0 | 0 | 0 | 0 | 1 (5.6) | 1 (5.6) | 1 (2.4) | 1 (2.4) |
| Infusion related reaction | 0 | 0 | 1 (5.9) | 1 (5.9) | 0 | 0 | 1 (2.4) | 1 (2.4) |
| Hypertension | 0 | 0 | 0 | 0 | 1 (5.6) | 1 (5.6) | 1 (2.4) | 1 (2.4) |

Data are n (%). Treatment-related adverse events of any grade occurring in more than 10% of total patients and all treatment-related adverse events of grade 3–5 were listed.

## Table S4. Serious adverse events

|  | 40/60 group (N=6) | 60/60 group (N=17) | 60/85 group (N=18) | Total (N=41) |
| --- | --- | --- | --- | --- |
| **Serious adverse events** | 3 (50.0) | 8 (47.1) | 8 (44.4) | 19 (46.3) |
| Neutrophil count decreased | 1 (16.7) | 1 (5.9) | 1 (5.6) | 3 (7.3) |
| White blood cell count decreased | 1 (16.7) | 1 (5.9) | 0 | 2 (4.9) |
| Pneumonia | 0 | 0 | 2 (11.1) | 2 (4.9) |
| Infection | 0 | 1 (5.9) | 1 (5.6) | 2 (4.9) |
| Upper gastrointestinal haemorrhage | 0 | 1 (5.9) | 1 (5.6) | 2 (4.9) |
| Intestinal obstruction | 0 | 1 (5.9) | 1 (5.6) | 2 (4.9) |
| Hypokalaemia | 0 | 0 | 1 (5.6) | 1 (2.4) |
| Eating disorder | 0 | 1 (5.9) | 0 | 1 (2.4) |
| Pyrexia | 0 | 1 (5.9) | 0 | 1 (2.4) |
| Death of unknown cause | 1 (16.7) | 0 | 0 | 1 (2.4) |
| Infusion related reaction | 0 | 1 (5.9) | 0 | 1 (2.4) |
| Alanine aminotransferase increased | 0 | 1 (5.9) | 0 | 1 (2.4) |
| Aspartate aminotransferase increased | 0 | 1 (5.9) | 0 | 1 (2.4) |
| Bilirubin conjugated increased | 0 | 1 (5.9) | 0 | 1 (2.4) |
| Bile acids increased | 0 | 1 (5.9) | 0 | 1 (2.4) |
| Blood bilirubin increased | 0 | 1 (5.9) | 0 | 1 (2.4) |
| COVID-19 pneumonia | 0 | 0 | 1 (5.6) | 1 (2.4) |
| Hepatorenal failure | 0 | 0 | 1 (5.6) | 1 (2.4) |
| Hepatic function abnormal | 1 (16.7) | 0 | 0 | 1 (2.4) |
| Jaundice cholestatic | 0 | 1 (5.9) | 0 | 1 (2.4) |
| Bile duct stenosis | 1 (16.7) | 0 | 0 | 1 (2.4) |
| Diarrhoea | 0 | 1 (5.9) | 0 | 1 (2.4) |
| Anaemia | 0 | 1 (5.9) | 0 | 1 (2.4) |
| Embolism | 0 | 0 | 1 (5.6) | 1 (2.4) |
| **Treatment-related serious adverse event** | 1 (16.7) | 5 (29.4) | 2 (11.1) | 8 (19.5) |
| Neutrophil count decreased | 1 (16.7) | 1 (5.9) | 1 (5.6) | 3 (7.3) |
| White blood cell count decreased | 1 (16.7) | 1 (5.9) | 0 (0.0) | 2 (4.9) |
| Infection | 0 (0.0) | 1 (5.9) | 1 (5.6) | 2 (4.9) |
| Intestinal obstruction | 0 (0.0) | 1 (5.9) | 1 (5.6) | 2 (4.9) |
| Eating disorder symptom | 0 (0.0) | 1 (5.9) | 0 (0.0) | 1 (2.4) |
| Infusion related reaction | 0 (0.0) | 1 (5.9) | 0 (0.0) | 1 (2.4) |
| Diarrhoea | 0 (0.0) | 1 (5.9) | 0 (0.0) | 1 (2.4) |
| Anaemia | 0 (0.0) | 1 (5.9) | 0 (0.0) | 1 (2.4) |
| Embolism | 0 (0.0) | 0 (0.0) | 1 (5.6) | 1 (2.4) |

Data are n (%). A total of three deaths due to adverse events were reported, including deaths of unknown cause, pneumonia, and hepatorenal failure, all of which are listed in this serious adverse events table. None of the three deaths were considered treatment-related.

## Table S5. Antitumor activity

|  | 40/60 group (N=6) | 60/60 group (N=17) | 60/85 group (N=18) | Total (N=41) |
| --- | --- | --- | --- | --- |
| Confirmed best overall response |  |  |  |  |
| Complete response | 0 | 0 | 0 | 0 |
| Partial response | 2 (33.3) | 4 (23.5) | 4 (22.2) | 10 (29.3) |
| Stable disease | 1 (16.7) | 8 (47.1) | 9 (50.0) | 18 (43.9) |
| Progression disease | 1 (16.7) | 3 (17.6) | 4 (22.2) | 8 (19.5) |
| Not evaluable | 2 (33.3) | 2 (11.8) | 1 (5.6) | 5 (12.2) |
| Confirmed objective response | 2 (33.3, 4.3–77.7) | 4 (23.5, 6.8–49.9) | 4 (22.2, 6.4–47.6) | 10 (24.4, 12.4–40.3) |
| Disease control | 3 (50.0, 11.8–88.2) | 12 (70.6, 44.0–89.7) | 13 (72.2, 46.5–90.3) | 28 (68.3, 51.9–81.9) |
| Duration of response, months | NR (3.9–NR) | 5.7 (3.4–NR) | 5.3 (2.0–13.2) | 5.5 (2.0–13.2) |
| Progression-free survival, months | NR (1.9–NR) | 5.4 (2.8–7.6) | 3.9 (2.2–7.2) | 5.4 (3.7–7.2) |
| Overall survival, months | 15.3 (2.4–27.9) | 10.3 (6.8–12.6) | 9.5 (6.5–11.2) | 10.3 (8.2–11.2) |

Data are n (%), n (%, 95% CI), or median (95% CI). NR, not reached.
